# The Geriatric Nutritional Risk Index (GNRI) as a Prognostic Biomarker for Immune Checkpoint Inhibitor Response in Recurrent and/or Metastatic Head and Neck Cancer

**DOI:** 10.3390/nu15040880

**Published:** 2023-02-09

**Authors:** Markus Haas, Alexander Lein, Thorsten Fuereder, Faris F. Brkic, Julia Schnoell, David T. Liu, Lorenz Kadletz-Wanke, Gregor Heiduschka, Bernhard J. Jank

**Affiliations:** 1 Department of Otorhinolaryngology and Head and Neck Surgery, Medical University of Vienna, 1090 Vienna, Austria; 2 Division of Oncology, Department of Medicine I, Medical University of Vienna, 1090 Vienna, Austria

**Keywords:** malnutrition, nutrition, squamous cell carcinoma, head and neck cancer, immune checkpoint inhibitor, immunotherapy, treatment response, geriatric nutritional risk index (GNRI)

## Abstract

Malnutrition is a frequent comorbidity in head and neck cancer patients and has been shown to impair immunotherapy response in other cancer types. The geriatric nutritional risk index (GNRI) assesses malnutrition using the patient’s ideal weight, actual weight, and serum albumin. The aim of this study was to evaluate the prognostic relevance of malnutrition as determined by the GNRI for the response to immunotherapy in recurrent and/or metastatic head and neck squamous cell carcinoma (R/M HNSCC). A total of 162 patients with R/M HNSCC who received immune checkpoint inhibitors were included. The associations between the GNRI and progression-free survival (PFS), overall survival (OS), and the disease control rate (DCR) were computed. Univariable analysis showed worse PFS for GNRI ≤ 98 (*p* < 0.001), ECOG performance status (PS) ≥ 2 (*p* = 0.012), and enteral (*p* = 0.009) and parenteral (*p* = 0.015) nutritional supplementation, and worse OS for GNRI < 92 (*p* < 0.001), ECOG PS ≥ 2 (*p* < 0.001), and enteral (*p* = 0.008) and parenteral (*p* = 0.023) nutritional supplementation. In our multivariable model, GNRI ≤ 98 (*p* = 0.012) and ECOG PS ≥ 2 (*p* = 0.025) were independent prognostic factors for PFS. For OS, GNRI < 92 (*p* < 0.001) and ECOG PS ≥ 2 (*p* < 0.001) were independent prognostic factors. A GNRI ≤ 98 was significantly associated with a lower DCR compared to a GNRI > 98 (*p* = 0.001). In conclusion, our findings suggest that the GNRI may be an effective predictor for response to immunotherapy in R/M HNSCC.

## 1. Introduction

In 2020, the International Agency for Research on Cancer estimated over 810,000 new cases and more than 414,000 deaths of squamous cell carcinoma of the head and neck (HNSCC) globally [1]. HNSCC can lead to dysphagia, dysgeusia, xerostomia, and oral mucositis, which can be caused directly by the primary tumor or due to treatment side effects, particularly from radio- and chemotherapy. These symptoms can severely impair daily calorie intake and are associated with increased mortality and morbidity [2,3]. Since a significant proportion of HNSCC patients require food supplements and tube feeding during treatment, managing their nutrition is an integral part of therapy. Especially in late-stage HNSCC, a multimodal treatment approach, including optimal nutritional interventions, is critical to improve patient outcome [4].

The current standard of care for patients with recurrent and/or metastatic head and neck squamous cell carcinoma (R/M HNSCC) is immune checkpoint inhibitor (ICI) treatment. Nivolumab and pembrolizumab, two monoclonal antibodies targeting the programmed cell death protein 1 (PD-1), received Food and Drug Administration approval in 2016 and 2019, respectively, for the palliative treatment of R/M HNSCC [5,6]. Interestingly, recent studies have shown that nutrition and diet can impact the efficacy of immunotherapy [7,8]. Therefore, identifying patients facing the risk of malnutrition plays a crucial role in the management of R/M HNSCC [9].

The geriatric nutritional risk index (GNRI) is based on weight, height, and serum albumin levels, making it easy to evaluate and implement in clinical routine [10]. It has been used to assess malnutritional risk in hospitalized adults over 65 years. Multiple studies have reported its prognostic impact in several cancer types, including HNSCC, independent of patient age [11,12,13]. Recently, Nakayama et al. reported a prognostic effect of the GNRI for overall survival in patients with advanced head and neck cancer at all ages [14]. To the best of our knowledge, the prognostic role of the GNRI in R/M HNSCC patients treated with immunotherapy has not yet been studied. Therefore, this study aimed to evaluate the GNRI as a prognosticator for progression-free survival (PFS), objective response, and overall survival (OS) in R/M HNSCC patients treated with ICI.

## 2. Materials and Methods

### 2.1. Study Population and Study Design

A total of 162 patients with R/M HNSCC who received nivolumab or pembrolizumab as palliative treatment between 2016 and 2021 at the Vienna General Hospital (Medical University of Vienna) were included in this study. Baseline characteristics and patient demographics were assessed prior to treatment initiation and retrospectively obtained from electronic medical records. History of heavy alcohol use was defined as having a previous medical record stating alcohol abuse as a comorbidity or stating self-reported daily drinking of more than three standard drinks per day [15]. Patients classified as smokers either had a reported history of tobacco use or were actively consuming tobacco at the time of treatment. Nutritional supplementation was reported as the primary route of administration at the time of ICI treatment initiation. Enteral supplementation included tube feeding via nasogastric tube or gastrostomy tube. Parenteral supplementation referred to total or partial parenteral nutrition. Serum albumin levels were assessed within the last 14 days before treatment initiation. Objective response was evaluated by CT and/or MRI studies according to Response Evaluation Criteria in Solid Tumors version 1.1 [16]. The disease control rate (DCR) was defined as the percentage of patients with complete response (CR), partial response (PR) or stable disease (SD) as their best overall response (bOR). The primary endpoint of this study was PFS, while OS and the DCR were secondary endpoints. The study was approved by the Ethics Committee of the Medical University of Vienna (EK1324/2022).

### 2.2. Score Calculation

The body-mass-index (BMI) was calculated as follows: weight(kg)/(height(m))^2^. Patients were assigned to one of four BMI categories according to World Health Organization standards [17]: underweight (BMI < 18.5 kg/m^2^), normal weight (BMI ≥ 18.5 kg/m^2^ ≤ 25.0 kg/m^2^), overweight (BMI ≥ 25.0 ≤ 30 kg/m^2^), obese (BMI ≥ 30.0 kg/m^2^). Ideal body weight was calculated via the Lorentz formula [10] for men: Ideal body weight (men) = height (cm) − 100 − ((height (cm) − 150)/4), and for women: Ideal body weight (women) = height (cm) − 100 − ((height (cm) − 150)/2). The GNRI was calculated according to Bouillanne et al. [10]: GNRI = (1.489 × serum albumin (g/l) + 41.7 × (present weight/ideal body weight). The present weight to ideal body weight ratio was set to 1 when the patient’s present weight exceeded the ideal body weight. Patients were assigned to one of four different malnutritional risk groups: no risk (GNRI > 98), low risk (GNRI 98–92), moderate risk (GNRI < 92–82), or major risk (GNRI < 82).

Based on our analysis for goodness of fit comparing four-group, three-group, and two-group GNRI models, we proposed dichotomized GNRI groups for PFS and OS. For PFS, the best model fit was identified for a two-group GNRI, resulting in GNRI > 98 (no risk) and GNRI ≤ 98 (at risk) subgrouping. For OS, the best model fit was identified for a two-group GNRI, resulting in GNRI ≥ 92 (no/low risk) and GNRI < 92 (moderate/major risk) subgrouping.

### 2.3. Statistical Analysis

All statistical analyses were performed using STATA (Version 14.0) and GraphPad PRISM (Version 9.0). Categorical variables are reported as number of patients (*n*) and percentage (%). PFS and OS were defined as the time between the first day of ICI administration and disease progression or death, respectively. Patients who were lost to follow-up were censored at the date of their last visit. The Kaplan–Meier estimator was used to generate survival curves. For survival analysis, hazard ratios were calculated using univariable and multivariable Cox proportional hazards models. In the multivariable model, we included all statistically significant results from the univariable analysis. To compare the goodness of fit between different GNRI models, we used the Akaike Information Criterion (AIC) in post hoc analysis. A smaller AIC value indicates a better model fit [18]. Differences in patient characteristics, demographics, bOR and DCR between two GNRI classifications were analyzed using the Pearson’s chi-squared test. The Fisher’s exact test was used for groups with one or more cell counts below five. All *p*-values ≤ 0.05 were considered statistically significant.

## 3. Results

### 3.1. Patient Characteristics and Demographics

The median age was 65 years (range 28–85). Regarding regimen, 82 patients (50.6%) received pembrolizumab, 33 patients (20.4%) received pembrolizumab + platinum + 5-fluorouracil (5-FU), and 47 patients (29.0%) received nivolumab.

In total, 80 (49.4%) patients scored a GNRI > 98 (no risk), while 82 (50.6%) patients had a GNRI ≤ 98 (at risk). Clinical and demographic characteristics of the study cohort are summarized in Table 1. There was a significant difference between BMI (*p* < 0.001), nutritional supplementation (*p* < 0.001), primary site (*p* = 0.031), p16 status (*p* = 0.046), and Eastern Cooperative Oncology Group performance status (ECOG PS) (*p* = 0.001) among the GNRI subgroups.

### 3.2. Survival

**Figure 1:**
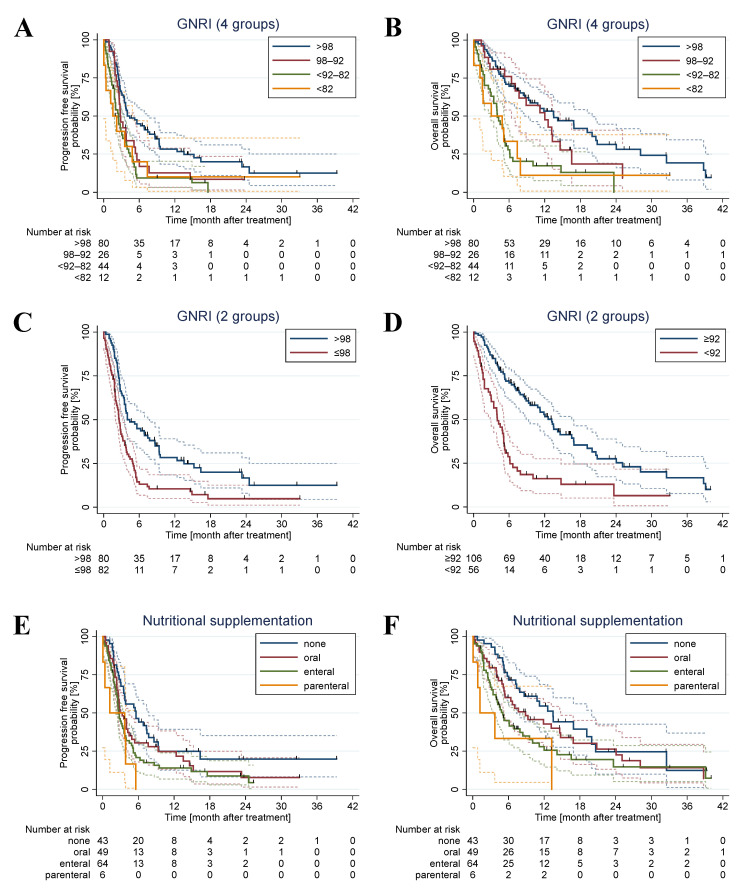
Survival analysis. Kaplan–Meier plots with 95% CI (dashed lines) of PFS and OS for GNRI (4 groups) (**A**,**B**), PFS and OS for GNRI (2 groups) cut off (**C**,**D**) and PFS and OS for nutritional supplementation (**E**,**F**). PFS, progression-free survival; OS, overall survival; GNRI, Geriatric Nutritional Risk Index.

The one-year PFS rate according to the four-group GNRI was 28.3% in the >98 (no risk) group, 12.7% in the 98–92 (low risk) group, 9.4% in the <92–82 (moderate risk) group, and 10.0% in the <82 (major risk) group (Figure 1A). The one-year OS rate in the >98 (no risk), 98–92 (low risk), <92–82 (moderate risk), and the <82 (major risk) group was 53.1%, 47.5%, 17.3%, and 11.1%, respectively (Figure 1B).

We analyzed different GNRI risk group models according to their goodness of fit. For PFS, AIC analysis of univariable Cox regression identified a two-group GNRI with >98 (no risk) and ≤98 (at risk) group as the best model fit (Table 2). The AIC analysis of univariable Cox regression for OS showed the best model fit for a two-group GNRI of ≥92 (no/low risk) and <92 (moderate/major risk) (Table 2).

The one-year PFS rate was significantly poorer in the GNRI ≤ 98 (at risk) group than in the GNRI > 98 (no risk) group (HR: 1.98; 95% CI: 1.41–2.80; *p* < 0.001) with a one-year survival rate of 10.5% and 28.3%, respectively (Figure 1C). For OS, the survival was significantly worse in the <92 (moderate/major risk) group compared to the ≥92 (no/low risk) group (HR: 2.74; 95% CI: 1.86–4.03; *p* < 0.001) with a one-year survival rate of 16.2% and 51.6%, respectively (Figure 1D).

Univariable Cox regression analysis of all patient characteristics showed that ECOG PS ≥ 2 was associated with shorter PFS (HR: 1.73; 95% CI: 1.13–2.65; *p* = 0.012) and shorter OS (HR: 2.53; CI: 1.59–4.01; *p* < 0.001) (Table 3). Additionally, nutritional supplementation significantly impacted survival. Enteral supplementation via nasogastric or gastrotomy tube significantly shortened PFS (HR: 1.79; 95% CI: 1.16–2.76; *p* = 0.009) and OS (HR: 1.92; 95% CI: 1.19–3.12; *p* = 0.008). Equivalently, parenteral supplementation reduced PFS (HR: 2.98; 95% CI: 1.24–7.17; *p* = 0.015) and OS (HR: 3.05; 95% CI: 1.16–7.98; *p* = 0.023). (Figure 1E,F)

Based on the results of the univariable analysis, we included ECOG PS and nutritional supplementation in our multivariable model. All results of the multivariable Cox regression analysis are shown in Table 4. Multivariable analysis was calculated for PFS and OS with the respective dichotomized and original GNRI. For PFS, according to dichotomized GNRI, GNRI ≤ 98 (HR: 1.65; 95% CI: 1.12–2.42; *p* = 0.012) and ECOG PS ≥ 2 (HR: 1.67; 95% CI: 1.07–2.63; *p* = 0.025) were independent prognostic factors. In the original GNRI model, the <92–82 (moderate risk) group (HR: 1.80; 95% CI: 1.15–2.81; *p* = 0.010) and ECOG PS ≥ 2 (HR: 1.72; 95% CI: 1.09–2.70; *p* = 0.019) were significant independent prognostic factors for PFS (Table 4). The multivariable analysis for OS with the dichotomized GNRI model, the GNRI < 92 (HR: 2.2; 95% CI: 1.45–3.35; *p* ≤ 0.001) and ECOG PS ≥ 2 (HR: 2.39; 95% CI: 1.48–3.85; *p* ≤ 0.001) were independent prognostic factors. In the four-group GNRI model, the <92–82 (moderate risk) group ((HR: 2.25; 95% CI: 1.38–3.69; *p* = 0.001), the < 82 (major risk) group (HR: 2.09; 95% CI: 1.02–4.27; *p* = 0.045), and ECOG PS ≥ 2 (HR: 2.39; 95% CI: 1.46–3.90; *p* = 0.001) were independent prognostic factors (Table 4).

In the AIC analysis of the multivariable models for PFS and OS (Table 4), the respective two-group GNRI models showed better model fit than the respective four-group GNRI model.

### 3.3. Best Overall Response

**Figure 2:**
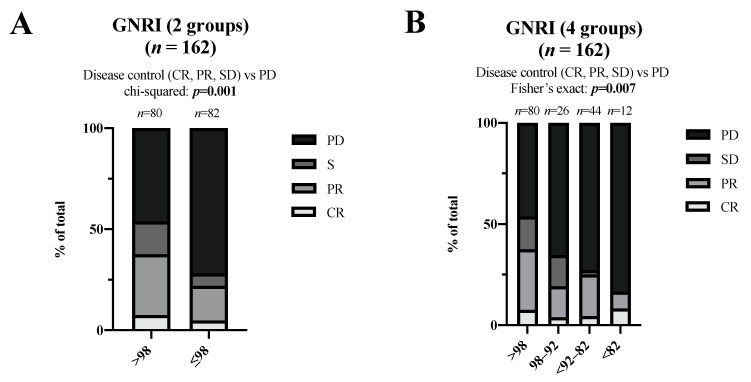
bOR of dichotomized GNRI (**A**) and original GNRI (**B**) groups. The difference in DCR (CR, PR, or SD) between groups was compared using chi-squared or Fisher’s exact test. Significant results (*p* ≤ 0.05) are highlighted in bold print. GNRI, Geriatric Nutritional Risk Index; DCR, disease control rate; CR, complete response; PR, partial response; PD, progressive disease; SD, stable disease.

Next, we analyzed the bOR and DCR in the dichotomized GNRI and the original four-group GNRI. According to the dichotomized GNRI, the DCR in the >98 (no risk) group was 54.0% and in the ≤ 98 (at risk) group 28.0%. The bOR in the >98 group showed 6 (7.5%) patients with CR, 24 (30%) patients with PR, 18 (16.3%) patients with SD, and 96 (46.3%) patients with progressive disease (PD). In the ≤ 98 group, patient numbers for CR, PR, SD, and PD were 4 (4.9%), 14 (17.1%), 5 (6.1%), and 59 (72.0%), respectively. The DCR was significantly higher (*p* = 0.001) in the >98 (no risk) group compared to the ≤ 98 (at risk) group (Figure 2A).

Among the four-group GNRI, the disease control rate (DCR) in the GNRI >98 (no risk), 98–92 (low risk), <92–82 (moderate risk), and the <82 (major risk) group was 53.8%, 34.6%, 27.3%, and 16.7%, respectively. The DCR of the GNRI >98 (no risk) group was significantly higher compared to the GNRI 98–92 (low risk), the GNRI < 92–82 (moderate risk), and the GNRI < 82 (major risk) group (Fisher’s exact: *p* = 0.007) (Figure 2B).

## 4. Discussion

In the current study, we examined the prognostic value of the GNRI for treatment response and survival in R/M-HNSCC patients receiving ICI treatment. To the best of our knowledge, this is the first retrospective cohort study to investigate GNRI predicting outcomes and ICI treatment response in HNSCC patients. We showed that a low GNRI was significantly associated with decreased PFS and OS. Moreover, we revealed divergent GNRI cut-off values for PFS and OS during analysis for goodness of model fit. In the multivariable analysis, we demonstrated that GNRI is an independent prognostic marker for PFS and OS. Patients with a GNRI > 98 had significantly higher DCR than patients with a GNRI ≤ 98.

It is estimated that up to 20% of cancer deaths are caused by malnutrition rather than the tumor itself [9]. Head and neck cancer patients are particularly susceptible to malnutrition, as it affects up to 67% of this patient population [19]. Malnutrition is associated with organ dysfunction, gut microbiota disturbance, and dysfunction in metabolic and immune pathways [20]. Furthermore, this leads to reduced disease resistance, decreased physical activity, and impaired wound healing, which are all associated with worse patient outcomes [21]. Therefore, it is crucial to identify and treat malnutrition in cancer patients using validated screening tools.

However, there is no standardized method to identify malnutrition. According to the European Society of Clinical Nutrition and Metabolism, a patient with a BMI < 18.5 can be classified as malnourished [22]. In the present study, our univariable analysis revealed that being underweight (BMI < 18.5) was no prognostic marker for PFS, OS or treatment response. The available literature on BMI as a prognostic marker for survival and treatment response in ICI patients presents heterogeneous results. While there is a plethora of data suggesting that a higher BMI is prognostically favorable in ICI treatment, the data on lower BMI (i.e., malnutrition) remain limited [23]. In addition, the use of different BMI cut-off values further limits comparability [24,25]. In line with our results, a cross-cancer study by Johannet et al. revealed that a BMI < 18.5 had no influence on PFS and OS [26]. In contrast, Zhang et al. showed that low BMI was an independent marker for a worse clinical prognosis in R/M HNSCC patients receiving pembrolizumab [27]. These results suggest that the BMI may not be optimal for defining malnutrition in cancer patients receiving ICI [17]. Moreover, the BMI has major limitations. It neglects metabolic changes, chronic diseases and weight loss and, therefore, fails to assess the complexity of the pathophysiological impact of malnutrition [17]. To overcome this problem, multiple nutritional assessment tools involving a variety of parameters have been proposed in the last decades [28]. Among these, the GNRI has emerged as a robust and easy-to-survey biomarker. First, the GNRI includes albumin, which has been shown to be a valuable biomarker for malnutrition, general health, and chronic inflammation [29,30]. Moreover, albumin decrease has been shown to be associated with rapid clearance of pembrolizumab in advanced cancer [31]. Second, the GNRI incorporates ideal weight into its calculation, which better resembles dynamic changes in chronic diseases such as tumor cachexia [26,27,32]. For example, Johannet et al. showed that malnutrition, defined as weight loss six months prior to treatment, had a negative impact on immunotherapy response in non-small cell lung cancer, while baseline BMI did not [26].

Our study showed that a low GNRI was significantly associated with worse PFS and OS in R/M HNSCC patients receiving immunotherapy. In our multivariable model, the GNRI and ECOG PS prevailed as independent prognostic factors for PFS and OS. The predictive effect of ECOG PS on survival was shown in large prospective studies. Next to PD-L1, it is the most used predictive factor in immunotherapy response [33]. Moreover, the association of low GNRI with worse OS is consistent with the results reported in the literature. For example, a study by Yamahara et al. showed that low GNRI was associated with worse OS in a cohort of 164 patients with advanced head and neck cancer [34]. Similarly, Nakayama et al. found that low GNRI was correlated with significantly worse OS in a study population of 248 patients with advanced head and neck cancer [14]. More recently, Yamagata et al. reported that the GNRI was an independent prognostic marker for OS in a cohort of 162 patients with oral squamous cell carcinoma [12]. All mentioned studies used the GNRI independent of patient age. However, none of these studies have investigated the effect of GNRI on PFS or treatment response.

Another advantage of the GNRI is the ability to assess the severity of malnutrition through risk group stratification. Clinical relevance of this fact is demonstrated in our goodness of fit analysis. To the best of our knowledge, our study is the first to reveal different cut-off values for PFS and OS at GNRI > 98 and GNRI ≥ 92, respectively. These data imply that minor malnutrition may affect immunotherapy response, although it does not significantly impact overall survival probability. Minor imbalances or changes in nutritional intake impact immune cell function, impairing immunotherapy response while having little influence on the general state of health [35]. One may argue that according to its mechanism of action, immunotherapy is more susceptible to treatment failure through insufficient nutrition status than other anticancer drugs. However, there are currently no data available for head and neck cancer. Nevertheless, preserving a good nutritional status appears essential for promoting favorable immunotherapy response [36].

The present study showed a better DCR in patients with a GNRI > 98. In line with our results, the GNRI has demonstrated prognostic value in immunotherapy response in various other cancer types [33,37,38]. Our results provide additional evidence that malnutrition is significantly associated with ICI response in R/M HNSCC patients. The mechanisms underlying this association still need to be fully understood [39]. However, malnutrition likely impairs the function of immune cells, including T cells and natural killer cells, which are critical for the effectiveness of immunotherapy [40]. Another theory suggests that dietary intake and malnutrition alter gut microbiota which is strongly associated with immunotherapy response [41,42].

Given the link between malnutrition and response to ICI, it is essential for clinicians to assess and address the nutritional status of cancer patients receiving immunotherapy. This may include implementing nutritional support interventions such as nutrition education, enteral or parenteral nutrition, and supplements to improve the nutritional status of these patients [9,43]. Interestingly, enteral and parenteral nutritional supplementation were significant prognosticators during our univariable analysis but failed to reach significance in the multivariable model by a small margin. Additionally, patients at malnutrition risk, according to the GNRI, were more likely to receive enteral and parenteral nutritional support. R/M HNSCC patients have frequently undergone extensive surgical and/or (chemo)radiation treatment by the time ICI therapy is indicated. Tube feeding is, therefore, common in these patients who are inherently at risk for malnutrition due to the side effects of prior treatment. On the one hand, the prognostic effects of enteral and parenteral nutritional support status could simply be the result of its correlation with higher malnutrition risk in extensively pre-treated R/M HNSCC patients. However, non-oral nutritional support could also affect ICI response through other mechanisms, as enteral and parenteral nutrition can affect the gut microbiome [44,45]. Given the link between the microbiome and immunotherapy response [41,42], further investigation on the effects of nutritional support on survival in RM/HNSCC patients receiving ICI is warranted.

Our study had several limitations. First, we retrospectively assessed patient characteristics and demographics at a single institution. Due to the limited data available in this setting, we only used one nutritional status assessment tool. The GNRI could potentially be used in conjunction with other nutritional assessments to provide a more comprehensive picture of the nutritional status of cancer patients. However, its simple and readily available nature makes it suitable for the daily clinical routine. Furthermore, our study cohort consisted of patients receiving either nivolumab or pembrolizumab. Although the mechanisms of these two PD-1 inhibitors are the same, nivolumab is only approved in platinum-resistant R/M HNSCC, which face a particularly poor prognosis [6]. Lastly, the sample size in our study was relatively small, which may limit the generalizability of our findings. However, as the treatment with ICI for R/M HNSCC has only been clinically available for a few years, our sample size is comparable to other study cohorts in the current literature. Nevertheless, future prospective studies with larger cohorts and additional nutritional assessment tools are needed.

In conclusion, our data suggest that pretreatment GNRI is associated with immunotherapy response and survival in R/M HNSCC patients. The GNRI is an easy-to-assess tool and, therefore, may be implemented in clinical practice as an effective prognosticator for survival and immunotherapy response.

## Figures and Tables

**Table 1:** Patient characteristics and demographics according to PFS-optimized GNRI groups. Distribution of GNRI > 98 (no risk) and GNRI ≤ 98 (at risk) was analyzed using Chi-squared or Fisher’s exact test.

	Total		GNRI (>98) No Risk		GNRI (≤98) at Risk		
Variables/Categories	*n*	(%)	*n*	(%)	*n*	(%)	*p*-Value
**Number of patients**	162	(100.0%)	80	(49.4%)	82	(50.6%)	
**Age**							
≥65	76	(46.9%)	43	(53.8%)	43	(52.4%)	
<65	86	(53.1%)	37	(46.3%)	39	(47.6%)	0.867
**Sex**							
male	115	(71.0%)	62	(77.5%)	53	(64.6%)	
female	47	(29.0%)	18	(22.5%)	29	(35.4%)	0.071
**BMI**							
underweight	36	(22.2%)	6	(7.5%)	30	(36.6%)	
normal weight	102	(63.0%)	50	(62.5%)	52	(63.4%)	
overweight	17	(10.5%)	17	(21.3%)	0	(0.0%)	
obese	7	(4.3%)	7	(8.8%)	0	(0.0%)	**<0.001**
**Nutritional supplementation**							
none	43	(26.5%)	35	(43.8%)	8	(9.8%)	
oral	49	(30.2%)	22	(27.5%)	27	(32.9%)	
enteral	64	(39.5%)	22	(27.5%)	42	(51.2%)	
parenteral	6	(3.7%)	1	(1.3%)	5	(6.1%)	**<0.001**
**History of heavy alcohol use**							
no	114	(70.4%)	56	(70.0%)	58	(70.7%)	
yes	48	(29.6%)	24	(30.0%)	24	(29.3%)	0.919
**History of smoking**							
no	11	(6.8%)	7	(8.8%)	4	(4.9%)	
yes	128	(79.0%)	62	(77.5%)	66	(80.5%)	0.366
unknown	23	(14.2%)	11	(13.8%)	12	(14.6%)	
**Primary site**							
oral cavity	66	(40.7%)	26	(32.5%)	40	(48.8%)	
oropharynx	33	(20.4%)	18	(22.5%)	15	(18.3%)	
hypopharynx	20	(12.3%)	7	(8.8%)	13	(15.9%)	
larynx	17	(10.5%)	11	(13.8%)	6	(7.3%)	
sinonasal	9	(5.6%)	3	(3.8%)	6	(7.3%)	
others ^§^	17	(10.5%)	15	(18.8%)	2	(2.4%)	**0.003**
**OPSCC (p16 positive)**							
yes	13	(8.0%)	10	(12.5%)	3	(3.7%)	
no	149	(92.0%)	70	(87.5%)	79	(96.3%)	**0.046**
**Disease extent**							
locoregional	75	(46.3%)	39	(48.8%)	36	(43.9%)	
distant metastasis	17	(10.5%)	8	(10.0%)	9	(11.0%)	
locoregional + distant metastasis	70	(43.2%)	33	(41.3%)	37	(45.1%)	0.826
**Prior primary treatment**							
surgical	26	(16%)	10	(12.5%)	16	(19.5%)	
surgical + poRT	33	(20.4%)	18	(22.5%)	15	(18.3%)	
surgical + poCRT/RIT	13	(8%)	7	(8.8%)	6	(7.3%)	
RT	19	(11.7%)	10	(12.5%)	9	(11%)	
CRT/RIT	58	(35.8%)	28	(35%)	30	(36.6%)	
palliative only	13	(8%)	7	(8.8%)	6	(7.3%)	0.862
**Prior palliative chemotherapy**							
yes	126	(77.8%)	66	(82.5%)	60	(73.2%)	
no	36	(22.2%)	14	(17.5%)	22	(26.8%)	0.153
**Regimen**							
Pembrolizumab	82	(50.6%)	47	(58.8%)	35	(42.7%)	
Pembrolizumab + platinum + 5-FU	33	(20.4%)	15	(18.8%)	18	(22.0%)	
Nivolumab	47	(29.0%)	18	(22.5%)	29	(35.4%)	0.101
**CPS**							
<1	3	(1.9%)	1	(1.3%)	2	(2.4%)	
1–20	48	(29.6%)	24	(30.0%)	24	(29.3%)	
>20	59	(36.4%)	34	(42.5%)	25	(30.5%)	0.538
unknown	52	(32.1%)	21	(26.3%)	31	(37.8%)	
**ECOG PS**							
0	73	(45.1%)	46	(57.5%)	27	(32.9%)	
1	52	(32.1%)	24	(30.0%)	28	(34.1%)	
≥2	37	(22.8%)	10	(12.5%)	27	(32.9%)	**0.001**

BMI, body mass index; 5-FU, 5-Fluorouracil; CPS, combined positive score; ECOG PS, Eastern Cooperative Oncology Group Performance Status; CR, complete response; OPSCC, oropharyngeal squamous cell carcinoma; po, postoperative; CRT, chemoradiotherapy; RIT, radioimmunotherapy; RT, radiotherapy; ^§^: carcinoma of unknown primary of the head and neck (*n* = 7), salivary glands (*n* = 3), cutaneous squamous cell carcinoma of the head and neck (*n* = 3), multifocal (*n* = 2), nasopharynx (*n* = 2). Significant results (*p* ≤ 0.05) are highlighted in bold print.

**Table 2:** AIC analysis of univariable Cox regression for PFS and OS of seven GNRI cut-off variants.

		PFS				OS			
GNRI	Level	HR	(95% CI)	*p*-Value	AIC	HR	(95% CI)	*p*-Value	AIC
**2 groups**	>98 vs. ≤98	1.98	(1.41–2.80)	**<0.001**	**1162.90**	2.15	(1.47–3.13)	**<0.001**	961.92
**2 groups**	≥92 vs. <92	1.97	(1.39–2.81)	**<0.001**	1164.71	2.74	(1.86–4.03)	**<0.001**	**953.60**
**2 groups**	≥82 vs. <82	1.59	(0.83–3.03)	0.161	1176.42	1.98	(1.03–3.81)	**0.040**	974.32
**3 groups**	>98 vs. 98–82	1.96	(1.37–2.80)	**<0.001**	1164.85	2.06	(1.39–3.05)	**<0.001**	963.29
	>98 vs. <82	2.11	(1.08–4.14)	**0.029**	-	2.74	(1.38–5.45)	**0.004**	-
**3 groups**	>98 vs. 98–92	1.61	(0.99–2.61)	0.053	1163.24	1.30	(0.75–2.24)	0.346	954.74
	>98 vs. <92–82	2.21	(1.52–3.23)	**<0.001**	-	2.92	(1.93–4.42)	**<0.001**	-
**3 groups**	≥92 vs. 92–82	2.00	(1.37–2.91)	**<0.001**	1166.69	2.78	(1.83–4.21)	**<0.001**	955.57
	≥92 vs. <82	1.90	(0.98–3.67)	0.057	-	2.61	(1.33–5.11)	**0.005**	
**4 groups**	>98 vs. 98–92	1.61	(0.99–2.61)	0.053	1165.22	1.30	(0.75–2.24)	0.345	956.71
	>98 vs. <92–82	2.24	(1.50–3.35)	**<0.001**	-	2.97	(1.91–4.62)	**<0.001**	-
	>98 vs. <82	2.11	(1.08–4.14)	**0.029**	-	2.78	(1.40–5.53)	**0.004**	-

PFS, progression-free survival; OS, progression-free survival; GNRI, geriatric nutritional risk index; HR, hazard ratio; CI, confidence interval; AIC, Akaike Information Criterion. Significant results (*p* ≤ 0.05) and lowest AIC value are highlighted in bold print.

**Table 3:** Univariable Cox regression for PFS and OS of all patient characteristics and demographics.

			PFS			OS	
Variables/Levels	*n*	HR	(95% CI)	*p*-Value	HR	(95% CI)	*p*-Value
**Age**	162						
≥65 vs. <65 (ref)		1.04	(0.74–1.47)	0.800	1.26	(0.87–1.83)	0.214
**Sex**	162						
female vs. male (ref)		1.10	(0.76–1.59)	0.628	1.11	(0.74–1.67)	0.608
**BMI**	162						
underweight vs. normal weight (ref)		1.13	(0.75–1.70)	0.561	1.37	(0.87–2.15)	0.178
overweight vs. normal weight (ref)		0.69	(0.37–1.18)	0.161	1.04	(0.57–1.87)	0.908
obese vs. normal weight (ref)		0.50	(0.20–1.25)	0.138	0.51	(0.18–1.40)	0.190
**Nutritional supplementation**	162						
oral vs. none (ref)		1.43	(0.90–2.28)	0.134	1.34	(0.8–2.22)	0.264
enteral vs. none (ref)		1.79	(1.16–2.76)	**0.009**	1.92	(1.19–3.12)	**0.008**
parenteral vs. none (ref)		2.98	(1.24–7.17)	**0.015**	3.05	(1.16–7.98)	**0.023**
**History of heavy alcohol use**	162						
yes vs. no (ref)		0.91	(0.63–1.32)	0.629	1.24	(0.84–1.84)	0.281
**History of smoking**	139						
ever vs. never (ref)		0.68	(0.35–1.31)	0.250	1.87	(0.76–4.62)	0.175
**Primary site**	162						
oral cavity vs. larynx (ref)		1.23	(0.70–2.18)	0.473	1.29	(0.67–2.47)	0.451
oropharynx vs. larynx (ref)		0.90	(0.47–1.69)	0.734	0.82	(0.40–1.69)	0.588
hypopharynx vs. larynx (ref)		1.15	(0.58–2.29)	0.684	0.58	(0.24–1.37)	0.212
sinonasal vs. larynx (ref)		1.12	(0.48–2.66)	0.789	1.05	(0.42–2.63)	0.912
other ^§^ vs. larynx (ref)		0.63	(0.29–1.34)	0.228	0.51	(0.22–1.20)	0.124
**OPSCC (p16 positive)**	162						
yes vs. no (ref)		1.01	(0.56–1.84)	0.961	0.91	(0.47–1.74)	0.766
**Disease extent**	162						
distant metastasis vs locoregional (ref)		0.65	(0.35–1.20)	0.165	0.48	(0.22–1.06)	0.070
locoregional + distant metastasis vs locoregional (ref)		0.77	(0.54–1.10)	0.142	0.93	(0.63–1.36)	0.699
**Prior primary treatment**	162						
surgical + poRT vs. surgical (ref)		1.24	(0.69–2.22)	0.475	1.31	(0.7–2.45)	0.402
surgical + poCRT/RIT vs. surgical (ref)		1.59	(0.77–3.28)	0.211	1.19	(0.53–2.68)	0.670
RT vs. surgical (ref)		1.45	(0.74–2.83)	0.276	0.98	(0.45–2.14)	0.955
CRT/RIT vs. surgical (ref)		1.30	(0.76–2.21)	0.337	1.34	(0.76–2.34)	0.309
palliative only vs. surgical (ref)		0.74	(0.34–1.64)	0.460	0.79	(0.35–1.79)	0.577
**Prior palliative chemotherapy**	162						
yes vs. no (ref)		1.23	(0.83–1.83)	0.306	1.11	(0.73–1.69)	0.628
**Regimen**	162						
Pembrolizumab + platinum + 5-FU vs Pembrolizumab (ref)		0.67	(0.41–1.08)	0.101	0.86	(0.51–1.47)	0.591
Nivolumab vs Pembrolizumab (ref)		1.35	(0.92–1.99)	0.120	1.27	(0.84–1.92)	0.250
**CPS score**	110						
1–20 vs. <1 (ref)		0.64	(0.19–2.08)	0.454	0.67	(0.16–2.84)	0.590
>20 vs. <1 (ref)		0.66	(0.20–2.14)	0.491	0.76	(0.18–3.18)	0.704
**ECOG**	162						
1 vs. 0 (ref)		1.15	(0.78–1.71)	0.482	1.15	(0.77–1.87)	0.409
≥2 vs. 0 (ref)		1.73	(1.13–2.65)	**0.012**	2.53	(1.59–4.01)	**<0.001**

BMI, body mass index; 5-FU, 5-Fluorouracil; CPS, combined positive score; ECOG, Eastern Cooperative Oncology Group; OPSCC, oropharyngeal squamous cell carcinoma; po, postoperative; CRT, chemoradiotherapy; RIT, radioimmunotherapy; RT, radiotherapy; PFS, progression-free survival; OS, progression-free survival; HR, hazard ratio; CI, confidence interval; ^§^: carcinoma of unknown primary of the head and neck (*n* = 7), salivary glands (*n* = 3), cutaneous squamous cell carcinoma of the head and neck (*n* = 3), multifocal (*n* = 2), nasopharynx (*n* = 2). Significant results (*p* ≤ 0.05) are highlighted in bold print.

**Table 4:** Multivariable Cox regression for PFS, OS and respective AIC analysis.

		PFS				OS			
Variables/Levels	*n*	HR	(95% CI)	*p*-Value	AIC	HR	(95% CI)	*p*-Value	AIC
**GNRI (2 groups)**	162								
≤98 vs. >98 (ref)		1.65	(1.12–2.42)	**0.** **012**	**1163.** **76**	-	-	-	-
<92 vs. ≥92 (ref)		-	-	-	-	2.20	(1.45–3.35)	**< 0.001**	**948.42**
**ECOG**	162								
1 vs. 0 (ref)		1.02	(0.67–1.54)	0.926	-	1.17	(0.74–1.84)	0.495	-
≥2 vs. 0 (ref)		1.67	(1.07–2.63)	**0.** **025**	-	2.39	(1.48–3.85)	**<** **0.001**	-
**Nutritional** **supplementation**	162								
oral vs. none (ref)		1.14	(0.69–1.87)	0.610	-	1.28	(0.77–2.14)	0.345	-
enteral vs. none (ref)		1.57	(0.98–2.52)	0.063	-	1.54	(0.92–2.58)	0.099	-
parenteral vs. none (ref)		2.45	(0.96–6.23)	0.060	-	2.65	(0.97–7.23)	0.056	-
**GNRI (4 groups)**	162								
98–92 vs. >98 (ref)		1.32	(0.77–2.28)	0.310	1166.40	1.01	(0.55–1.86)	0.980	952.38
<92–82 vs. >98 (ref)		1.80	(1.15–2.81)	**0.** **010**	-	2.25	(1.38–3.69)	**0.** **001**	-
<82 vs. >98 (ref)		1.84	(0.93–3.63)	0.081	-	2.09	(1.02–4.27)	**0.045**	-
**ECOG**	162								
1 vs. 0 (ref)		1.05	(0.69–1.59)	0.836	-	1.16	(0.73–1.85)	0.526	-
≥2 vs. 0 (ref)		1.72	(1.09–2.7)	**0.** **019**	-	2.39	(1.46–3.9)	**0.001**	-
**Nutritional** **supplementation**	162								
oral vs. none (ref)		1.22	(0.74–2.03)	0.438	-	1.28	(0.73–2.24)	0.383	-
enteral vs. none (ref)		1.54	(0.95–2.48)	0.080	-	1.53	(0.91–2.59)	0.111	-
parenteral vs. none (ref)		2.52	(0.98–6.45)	0.055	-	2.61	(0.92–7.42)	0.072	-

GNRI, geriatric nutritional risk score; ECOG, Eastern Cooperative Oncology Group; PFS, progression-free survival; OS, progression-free survival; GNRI, geriatric nutritional risk index; HR, hazard ratio; CI, confidence interval; AIC, Akaike Information Criterion. Significant results (*p* ≤ 0.05) and lowest AIC value are highlighted in bold print.

## Data Availability

Data are available from authors upon reasonable request.

## Abbreviations

HNSCC, head and neck squamous cell carcinoma; R/M HNSCC, recurrent and/or metastatic head and neck squamous cell carcinoma; ICI, immune checkpoint inhibitor; PD-1, programmed cell death protein 1; GNRI, geriatric nutritional risk index; DCR, disease control rate; CR, complete response; PR, partial response; SD, stable disease; bOR, best overall response; BMI, body-mass-index; AIC, Akaike Information Criterion; ECOG PS, Eastern Cooperative Oncology Group performance status; 5-FU, 5-fluorouracil
